# Compound Composite Odontoma and Its Management

**DOI:** 10.1155/2014/107089

**Published:** 2014-12-21

**Authors:** Morawala Abdul, Kapila Pragati, Chunawala Yusuf

**Affiliations:** ^1^Pedodontics & Preventive Dentistry, M.A. Rangoonwala College of Dental Sciences & Research Centre, Pune, Maharashtra 411001, India; ^2^Advanced Program for Dentists in Pediatric Dentistry, New York University College of Dentistry, New York, USA

## Abstract

Odontomas are odontogenic benign tumors composed of dental tissue. Majority of these lesions are asymptomatic and are often detected on routine radiographs. They can be thought of as “tooth hamartomas" with the lesion consisting of various tooth components. They are divided histologically into complex odontomas and compound odontomas. This paper describes the case of a compound odontoma in a 13-year-old girl diagnosed due to the retention of the primary right mandibular second molar. A surgical excision was performed without disturbing the unerupted premolar. The results achieved indicate that early diagnosis of odontomas enables adoption of less complex treatment, a better prognosis, and displacement or devitalisation of adjacent tooth.

## 1. Introduction

Odontomas are the most common odontogenic tumors of the jaws, characterized by their slow growth and nonaggressive behavior [1]. The term odontoma was first coined by Broca in 1866, who defined it as a tumor of overgrowth of complete dental tissue [2].

Odontomas are considered to be developmental anomalies resulting from the growth of completely differentiated epithelial and mesenchymal cells that give rise to ameloblasts and odontoblasts [1]. They are formed of enamel and dentin but can also contain variable amounts of cementum and pulp tissue [3, 4]. Odontomas are believed to be hamartomas and not true neoplasms because the epithelial and mesenchymal cells and tissues of an odontoma can appear normal but are deficient in structural arrangement [5].

The etiology of odontoma is still unclear [1]. Local traumas or infections may cause odontomas [6]. Radiographically, odontomas appear as dense radioopaque lesions with prominent external margins surrounded by a thin radiolucent zone [7, 8].

The World Health Organization (WHO) 2005 has classified odontomas according to the histopathological findings: complex odontomas, in which the dental tissues are well formed but exhibit an amorphous and more or less disorderly arrangement, and the compound odontomas, in which the dental tissues are normal, arranged in an orderly pattern, but their size and conformation are altered, giving rise to multiple small teeth like elements called odontoids or denticles [9].

The majority of odontomas which are located in the anterior region of the maxilla are compound, while the great majority of odontomas located in the posterior areas, especially in the mandible, are of the complex types [1].

Management consists of excision. Prognosis after treatment is very favorable, with scant relapse [1, 10, 11].

## 2. Case Report

**Figure 1:**
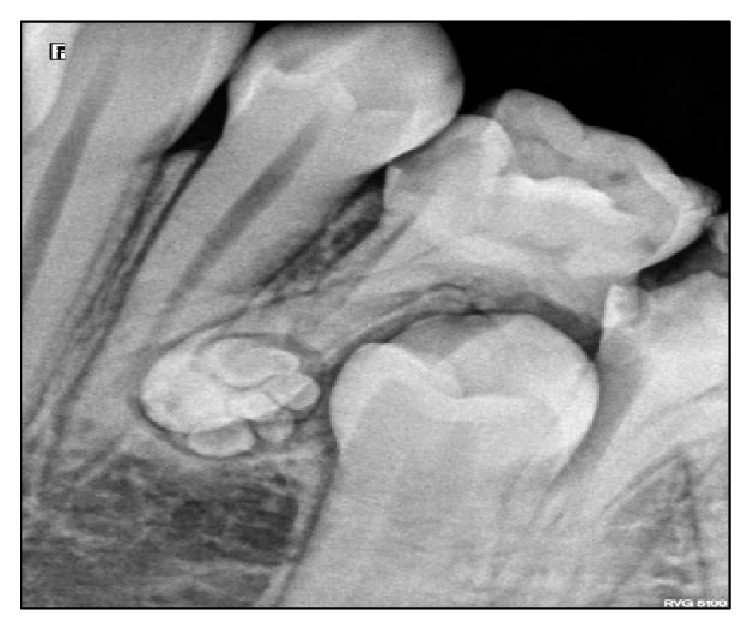


**Figure 2:**
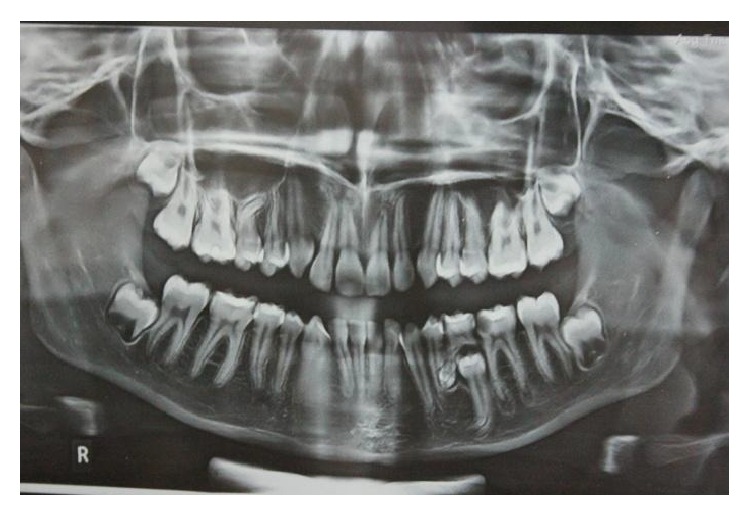


A 13-year-old female patient reported to the Department of Pedodontics with chief complaint of pain in mandibular right posterior region. Patient's medical and family history were inconclusive. Intraoral examination revealed the presence of occlusal caries on mandibular primary right second molar. The radiographic examination revealed the presence of occlusal caries involving enamel, dentin and approaching pulp Figures 1 and 2. There were multiple small teeth like radioopaque structures at the apex of distal root of mandibular right primary second molar. They were surrounded by a thin radiolucent zone measuring approximately 1.5 × 1.0 cm.

An OPG revealed the same findings as those of the intraoral periapical radiograph Figure 2. Based on the clinical and radiographic examination, a provisional diagnosis of compound odontoma was made. Surgical excision of the lesion by curettage and radiographic follow-up were done.

**Figure 3:**
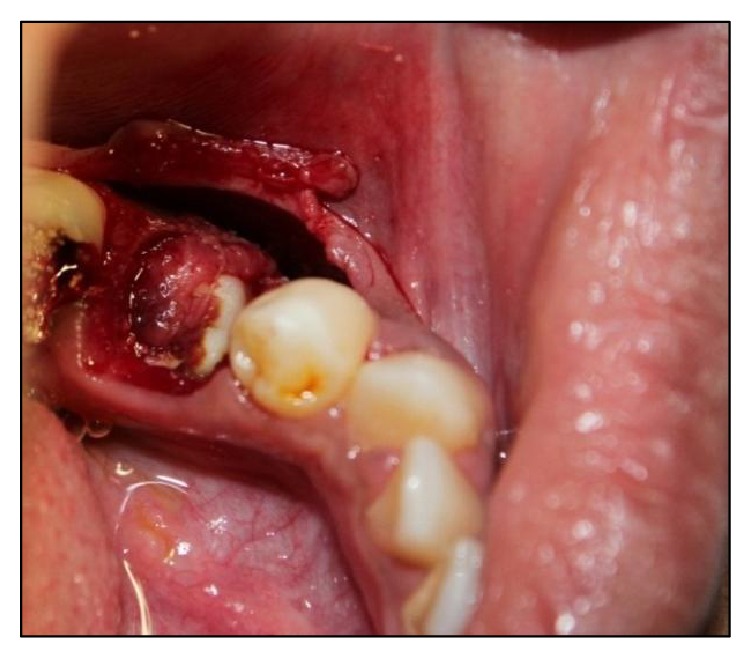


**Figure 4:**
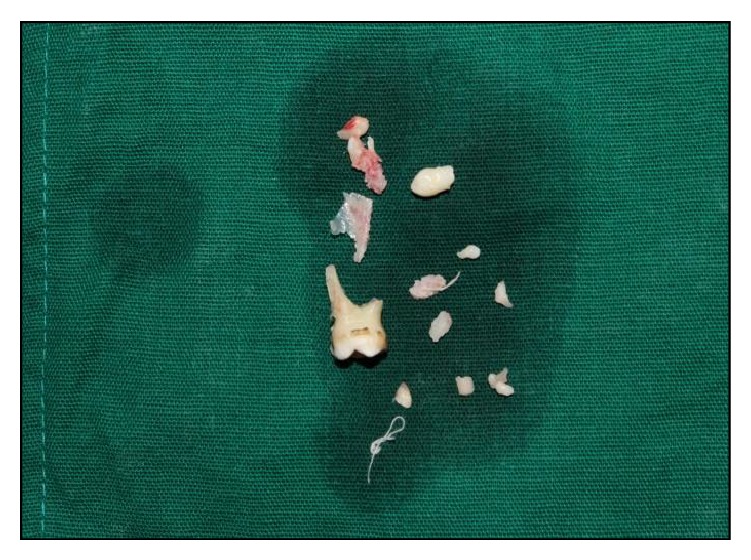


After achieving adequate local anaesthesia, a mucoperiosteal flap was reflected from the distal surface of the mandibular right primary first molar to the mesial surface of mandibular right permanent first molar on the labial surface. A layer of bone overlying the lesion was removed using a round surgical bur under constant irrigation with saline solution (Figure 3). The calcified teeth like structures were removed along with the fibrous capsule, without disturbing the unerupted permanent premolar (Figure 4).

**Figure 5:**
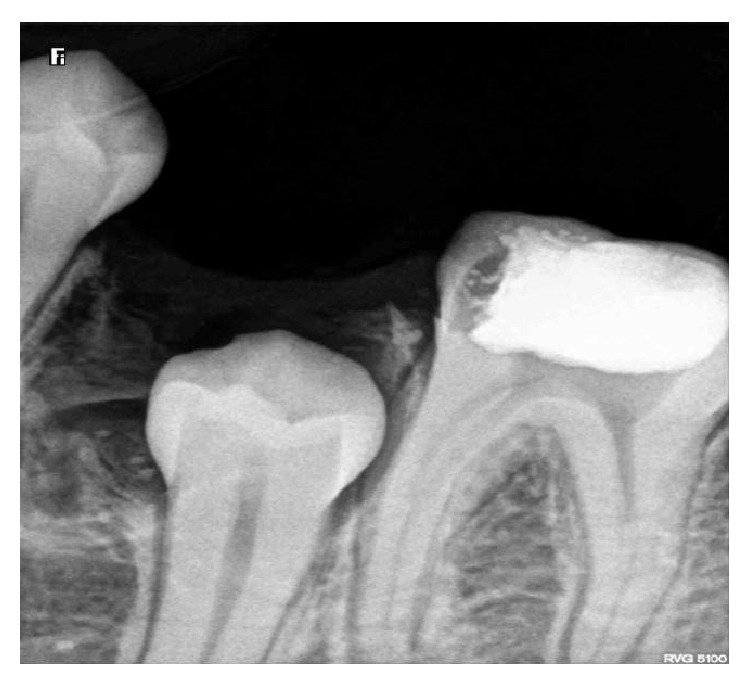


The surgical site was curetted and irrigated with povidone iodine-saline solution. After hemostasis was achieved, the flap was approximated and closed primarily with 3.0 silk sutures and postoperative radiograph was taken (Figure 5). Sutures were removed one week postoperatively. Histopathological examination confirmed the provisional diagnosis of compound odontoma, showing transverse and longitudinally cut dentinal tubules. Sections revealed are of amorphous basophilic masses resembling cementum. Dense fibrocellular connective tissue noted resembling periodontal ligament. Few sections also revealed numerous trabeculae with osteocytes in lacunae (Figure 7). These features are similar to those of odontomas which comprise varying amount of enamel and pulp tissue, enamel organ, and cementum. Odontogenic epithelium and mesenchymal pulp tissue also may present in some cases. The connective tissue capsule is similar to that of dental follicle. Ghost cells are often seen along with spherical dystrophic calcification, enamel, and sheets of dysplastic dentin [12–14].

**Figure 6:**
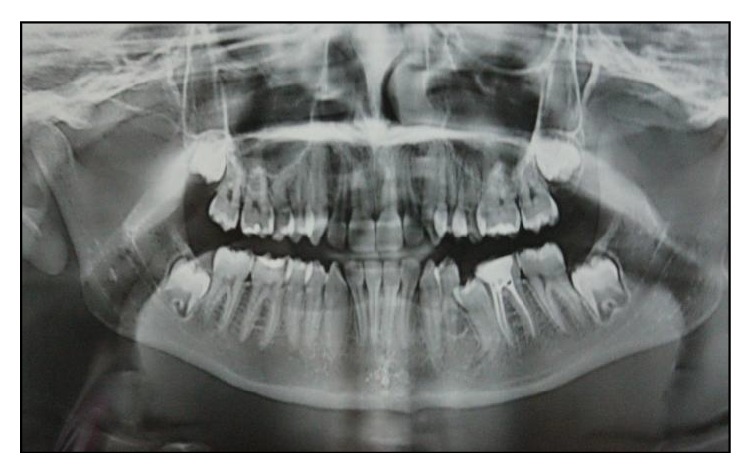


Six months postoperatively, OPG was advised to check for the eruption of second permanent premolar (Figure 6) and revealed a continuous eruption of the tooth into the oral cavity.

## 3. Discussion

### 3.1. Definition

Odontomas are nonaggressive, hamartomatous developmental malformations or lesions of odontogenic origin appearing as small, solitary, or multiple radiopaque lesions found on routine radiographic examinations [10]. They are the most common odontogenic tumors which constitute 22% of all the odontogenic tumors of the jaws [4].

### 3.2. Etiology

The etiology of odontomes remains unknown [1]. It has been associated with various pathological conditions, like local trauma, inflammatory and/or infectious processes, mature ameloblasts, cell rests of serres (dental lamina remnants) or hereditary anomalies (Gardner's syndrome and Hermann's syndrome), odontoblastic hyperactivity, and alterations in the genetic component responsible for controlling dental development [15].

### 3.3. Classification

In 1914, Gabell et al. grouped odontomes according to their developmental origin into: epithelial, composite (epithelial and mesodermal) and connective tissue [16, 17]. According to 2005 WHO classification of odontogenic tumours, there are two types of odontomas, compound and complex odontomas [9]. Clinically, they are classified as intraosseous (central), peripheral (soft tissue or extraosseous), and erupted odontomas. The central (intraosseous) odontomas are common representing 51%, occurring in anterior maxilla (compound odontoma) followed by mandibular molar region (complex odontoma). Peripheral odontomas are rare and occur in the soft tissue over the tooth-bearing zone and most reported ones are of compound variety. The erupted odontomas are the ones which are present coronal to an erupting or impacted tooth or superficially in bone and may have enabled its eruption into the oral cavity [18–20].

WHO defined a compound odontoma as a malformation in which all the dental tissues are represented in a more orderly pattern so that the lesion consists of many tooth like structures. Most of the structures do not morphologically resemble the teeth of the normal dentition but, in each one enamel, dentin, cementum, and pulp, are arranged as in the normal tooth [21]. Complex odontomes are seen less common in comparison with compound variety in the ratio 1 : 2 [22].

### 3.4. Incidence

The incidence of compound odontome ranges between 9% and 37%. The majority of odontomas in the anterior segment of the jaws are compound composite in type (61%), whereas the majority of odontomas in the posterior segment are complex composite in type (34%) [1, 22]. Interestingly, both types of odontomas occurred more frequently on the right side of the jaw than on the left, (compound 62% and complex 68%) [1, 3, 23]. The compound composite odontome most frequently occurred in incisor cuspid region of the upper jaw in contrast to the complex odontomes which were commonly found in molar and premolar region of the mandible [3, 6, 23, 24]. In contrast to our case, compound odontome located in posterior mandibular region which is quite rare condition.

Radiographic examination seems to be the most effective clinical method of differentiating between the two types [25, 26]. Compound odontoma, which radiographically shows comparatively a well-organized malformed teeth or tooth-like structures, usually is a radiolucent cyst like lesion whereas complex odontoma shows an irregularly shaped oval radiopacity usually surrounded by a well-defined thin radiolucent rim [26, 27].

**Figure 7:**
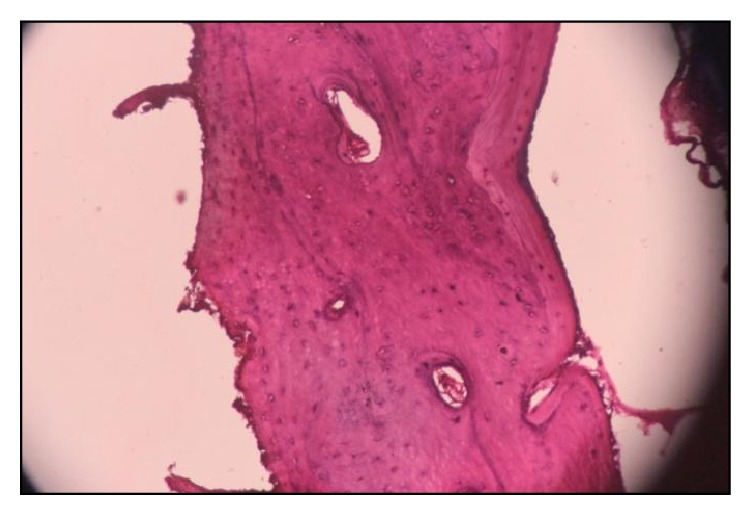


The case described in this report was initially diagnosed as compound odontoma based on the radiographic findings. This diagnosis was later confirmed by histopathologic examination of the lesion (Figure 7).

Odontomas, both compound and complex, must be examined microscopically to establish a definitive diagnosis [12, 14].

### 3.5. Treatment

Kaban states that odontomas are easily enucleated, and displaced adjacent teeth by the lesion are seldom traumatized during surgical excision because they are usually separated by a septum of bone [28]. An odontoma has a limited growth potential, but it should be removed because it contains various tooth formulations that can predispose to cystic change [29].

Thus, a thorough visual, manual, and radiographic examination should be performed for all the pediatric patients who present with clinical evidence of delayed eruption, missing tooth, or temporary tooth displacement, with or without history of trauma.

Early diagnosis of odontomas helps us to [18]

1. adopt a less complex and less expensive treatment,
2. ensure better prognosis,
3. avoid relapse of the lesion,
4. avoid displacement or devitalisation of adjacent tooth.

## References

[1] Shafer W. G., Hine M. K., Levy B. M. (1997). Cysts and tumours of the jaws. *A Textbook of Oral Pathology*.

[2] Cohen D. M., Bhattacharyya I. (2004). Ameloblastic fibroma, ameloblastic fibro-odontoma, and odontoma. *Oral & Maxillofacial Surgery Clinics of North America*.

[3] Philipsen H. P., Reichart P. A., Prætorius F. (1997). Mixed odontogenic tumours and odontomas. Considerations on interrelationship. Review of the literature and presentation of 134 new cases of odontomas. *European Journal of Cancer Part B: Oral Oncology*.

[4] Cuesta S. A., Albiol J. G., Aytés L. B., Escoda C. G. (2003). Review of 61 cases of odontoma. Presentation of an erupted complex odontoma. *Medicina Oral*.

[5] Shekar S. E., Roopa S. R., Gunasheela B., Supriya N. (2009). Erupted compound odontoma. *Journal of Oral and Maxillofacial Surgery*.

[6] Dagstan S., Goregen M., Miloglu Ö. (2007). Compound odontoma associated with maxillary impacted permanent central incisor tooth: a case report. *The Internet Journal of Dental Science*.

[7] Sprawson E. (1937). Odontomes. *British Dental Journal*.

[8] Bimstein E. (1978). Root dilaceration and stunting in two unerupted primary incisors. *ASDC Journal of Dentistry for Children*.

[9] Barnes L., Eveson J. W., Reichart P., Sidransky D. (2005). *World Health Organization Classification of Tumours. Pathology and Genetics of E558 Head and Neck Tumours*.

[10] Waldron A. C., Neville B. W. (2002). Odontogenic cysts and tumours. *Oral and Maxillofacial Pathology*.

[11] White S. C., Pharoah M. J. (2004). Benign tumours of the jaws. *Oral Radiology: Principles and Interpretation*.

[12] Bhasker S. N. (1977). *Synopsis of Oral Pathology*.

[13] Smith R. M., Tuner J. E., Ribbins M. L. (1981). *Atlas of Oral Pathology*.

[14] Wood N. K., Goaz P. W. (1985). *Differential Diagnosis of Oral Lesions*.

[15] Hitchin A. D. (1971). The aetiology of the calcified composite odontomes. *British Dental Journal*.

[16] Budnick S. D. (1976). Compound and complex odontomas. *Oral Surgery Oral Medicine and Oral Pathology*.

[17] Singh S., Singh M., Singh I., Khandelwal D. (2005). Compound composite odontome associated with an unerupted deciduous incisor—a rarity. *Journal of Indian Society of Pedodontics and Preventive Dentistry*.

[18] Satish V., Prabhadevi M. C., Sharma R. (2011). Odontome: a brief overview. *International Journal of Clinical Pediatric Dentistry*.

[19] Daley T. D., Wysocki G. P., Pringle G. A. (1994). Relative incidence of odontogenic tumors and oral and jaw cysts in a Canadian population. *Oral Surgery, Oral Medicine, Oral Pathology*.

[20] Ide F., Shimoyama T., Horie N. (2000). Gingival peripheral odontoma in an adult: case report. *Journal of Periodontology*.

[21] Pindborg J. J., Kramer I. R. H., Torloni H. (1970). *Histological Typing of Odontogenic Tumours, Jaw Cysts, and Allied Lesions*.

[22] Vengal M., Arora H., Ghosh S., Pai K. M. (2007). Large erupting complex odontoma: a case report. *Journal of the Canadian Dental Association*.

[23] Pindborg J. J., Hjortiy-Hansen E. (1974). *Atlas of Diseases of the Jaws*.

[24] Stajcic Z. Z. (1988). Odontoma associated with a primary tooth. *Journal of Pedodontics*.

[25] White S., Pharoah M. (2000). *Oral Radiology: Principles and Interpretation*.

[26] Stafne E. C., Giblisco J. A. (1975). *Oral Roentgenographic Diagnosis*.

[27] Goaz P. W., White S. C. (1987). *Oral Radiology*.

[28] Kaban L. B., Troulis M. J. (2004). Dentoalveolar surgery. *Pediatric Oral and Maxillofacial Surgery*.

[29] Kaban L. B. (1990). *Pediatric Oral and Maxillofacial Surgery*.

